# Categorization of 77 *dystrophin *exons into 5 groups by a decision tree using indexes of splicing regulatory factors as decision markers

**DOI:** 10.1186/1471-2156-13-23

**Published:** 2012-03-31

**Authors:** Rusdy Ghazali Malueka, Yutaka Takaoka, Mariko Yagi, Hiroyuki Awano, Tomoko Lee, Ery Kus Dwianingsih, Atsushi Nishida, Yasuhiro Takeshima, Masafumi Matsuo

**Affiliations:** 1 Department of Pediatrics, Graduate School of Medicine, Kobe University, Chuo, Kobe 6500017, Japan; 2 Division of Medical Informatics and Bioinformatics, Kobe University Hospital, Chuo, Kobe 6500017, Japan; 3 Department of Clinical Pharmacy, Kobe Pharmaceutical University, Higashinada, Kobe 6588558, Japan; 4 Department of Medical Rehabilitation, Faculty of Rehabilitation, Kobegakuin University, 518 Arise, Ikawadani, Nishi, Kobe 651-2180, Japan

**Keywords:** Splicing, *Dystrophin*, Exon, Splicing enhancer, Decision tree

## Abstract

**Background:**

Duchenne muscular dystrophy, a fatal muscle-wasting disease, is characterized by dystrophin deficiency caused by mutations in the *dystrophin *gene. Skipping of a target *dystrophin *exon during splicing with antisense oligonucleotides is attracting much attention as the most plausible way to express dystrophin in DMD. Antisense oligonucleotides have been designed against splicing regulatory sequences such as splicing enhancer sequences of target exons. Recently, we reported that a chemical kinase inhibitor specifically enhances the skipping of mutated *dystrophin *exon 31, indicating the existence of exon-specific splicing regulatory systems. However, the basis for such individual regulatory systems is largely unknown. Here, we categorized the *dystrophin *exons in terms of their splicing regulatory factors.

**Results:**

Using a computer-based machine learning system, we first constructed a decision tree separating 77 authentic from 14 known cryptic exons using 25 indexes of splicing regulatory factors as decision markers. We evaluated the classification accuracy of a novel cryptic exon (exon 11a) identified in this study. However, the tree mislabeled exon 11a as a true exon. Therefore, we re-constructed the decision tree to separate all 15 cryptic exons. The revised decision tree categorized the 77 authentic exons into five groups. Furthermore, all nine disease-associated novel exons were successfully categorized as exons, validating the decision tree. One group, consisting of 30 exons, was characterized by a high density of exonic splicing enhancer sequences. This suggests that AOs targeting splicing enhancer sequences would efficiently induce skipping of exons belonging to this group.

**Conclusions:**

The decision tree categorized the 77 authentic exons into five groups. Our classification may help to establish the strategy for exon skipping therapy for Duchenne muscular dystrophy.

## Background

Duchenne muscular dystrophy (DMD), a fatal muscle-wasting disease, is the most common inherited muscle disease, affecting one in every 3500 male births. DMD is characterized by dystrophin deficiency caused by mutations in the *dystrophin *gene, the largest human gene that spans over 2500 kb on the X-chromosome. For the treatment of DMD, antisense oligonucleotides (AOs) against splicing regulatory sequences have been proposed to produce in-frame *dystrophin *mRNA from the out-of-frame mRNA by inducing exon skipping during splicing [1]. The newly generated in-frame *dystrophin *mRNA is expected to produce semi-functional, internally deleted dystrophin protein. Currently, induction of exon skipping with AOs is considered one of the most promising treatments for DMD [2,3].

The *dystrophin *gene encodes a 14-kb mRNA consisting of 79 exons and is characterized by its huge intron size; the largest, intron 44, is 248 kb long. In addition, eight alternative promoters that are activated in a tissue-specific manner have been identified. Each tissue-specific exon 1 under the control of a cryptic promoter is spliced correctly to one of the downstream authentic *dystrophin *exons, producing a tissue-specific dystrophin isoform [4,5]. Furthermore, alternative splicings of some exons lead to the production of additional isoforms of the tissue-specific transcripts [6-8]. Remarkably, 14 cryptic exons that resemble authentic exons in terms of length and splice site strength, but are very rarely if ever spliced, have been reported within the huge introns [9,10].

Splicing is the process that removes introns from pre-mRNA, and is performed in the spliceosome, a ribonucleoprotein assembly. The spliceosome is one of the most complex cellular machineries, comprising approximately 150 proteins and five small nuclear RNAs (snRNAs U1, U2, U4, U5, and U6) [11]. Three sites, the splice donor site (5'ss), the splice acceptor site (3'ss), and the branch point sequences are the core splice site signals that are present in every intron. Despite the high potential for errors, the splicing process appears to occur with high fidelity, implying the widespread involvement of additional transcript features. These exonic elements are conventionally classified as exonic splicing enhancers (ESEs) or silencers (ESSs) and they function to promote or inhibit inclusion of the exon in which they reside, respectively. These splicing regulatory elements function by recruiting *trans*-acting splicing factors that activate or suppress splice site recognition or spliceosome assembly by various mechanisms [12]. AOs that induce skipping of *dystrophin *exons have been mainly designed against ESEs of target exons to hamper recruitment of splicing factors [2,3].

Recently, we reported that a small chemical enhances skipping of mutated *dystrophin *exon 31 in a sequence-specific manner, not altering the splicing of other *dystrophin *exons [13]. It was strongly suggested that *dystrophin *exons have their own splicing regulatory systems. However, the characteristics of the splicing regulatory systems of individual *dystrophin *exons are not well understood.

Decision trees are classifiers that predict class labels for data items and can make very accurate predictions [14,15]. They have been used to establish an integrated method that is one of the best available ways to find genes in the human genome [15].

Here, we identified a novel cryptic exon in intron 11 of the *dystrophin *gene in a DMD patient, and constructed decision trees to discriminate authentic exons from cryptic exons. Finally, we categorized 77 authentic exons into five groups based on indexes of their splicing regulatory factors. From the decision tree, we suppose that one group of exons showing high density for ESE sequences are a good target for exon skipping therapy.

## Results

**Figure 1:**
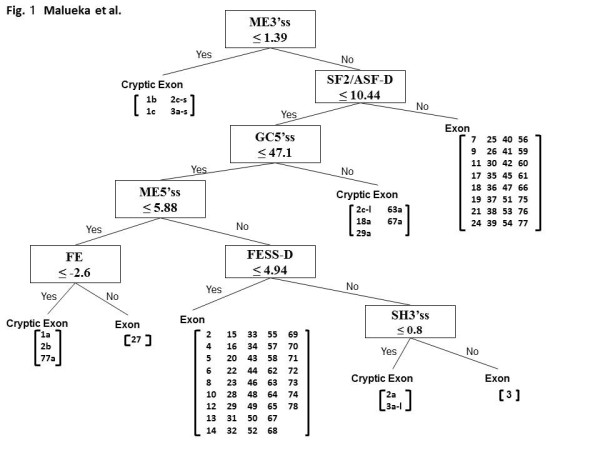
**Preliminary decision tree to classify 77 authentic and 14 cryptic *dystrophin *exons**. Exons are passed down the tree beginning at the top, where a "yes" result on any test means that it should be passed down to the left. The features tested in this tree are the maximum entropy at the 3' splice site (ss) (ME3'ss), the SF2/ASF density (SF2/ASF-D), the GC content at the 5'ss (GC5'ss), the maximum entropy at the 5'ss (ME5'ss), the free energy at the 5'ss U1 snRNP binding site (FE), the number of exonic splicing silencer (FESS), and the Shapiro score at the 3'ss (SH3'ss). The internal nodes of the tree represent index values that are tested for each exon as it is passed through the tree. Each successive node in the tree represents a decision that is based on those values, until a final classification is reached (the leaves). Authentic and cryptic exons were classified into four groups each.

To examine the splicing regulatory factors that characterize particular exons, we constructed decision trees classifying authentic from cryptic exons using indexes of splicing regulatory factors as decision markers. Cryptic exons within the *dystrophin *gene resemble authentic exons in terms of length and splice site strength, but are very rarely if ever spliced [10]. Therefore, analyzing the exon recognition parameters of these exons compared to the authentic *dystrophin *exons can give insight into which splicing regulatory elements actually play a critical role in the splicing of *dystrophin *exons. The goal of the decision tree was to determine the critical parameters that provided the most accurate categorization of authentic exons and cryptic exons. A preliminary decision tree was constructed to discriminate 77 authentic exons from 14 known cryptic exons. To classify these exons, we used 26 indexes that have been reported as important in proper splicing (see Methods). The decision tree system output a simple data structure (Figure 1). The decision tree revealed that the strength of the 3'ss calculated by maximum entropy (ME3'ss) was the first splitting variable, with a cut-off point of 1.39. At this node, four cryptic exons were classified into one group. At the next node, SF2/ASF-D was used as the splitting variable, with a cut-off point of less than 10.44, generating a group of 32 authentic exons. In this way, seven nodes were used to separate clearly the 77 authentic exons from the 14 cryptic exons. The authentic exons were categorized into four groups, comprising 43, 32, 1, and 1 exon, respectively; similarly, the cryptic exons were also categorized into four groups.

**Figure 2:**
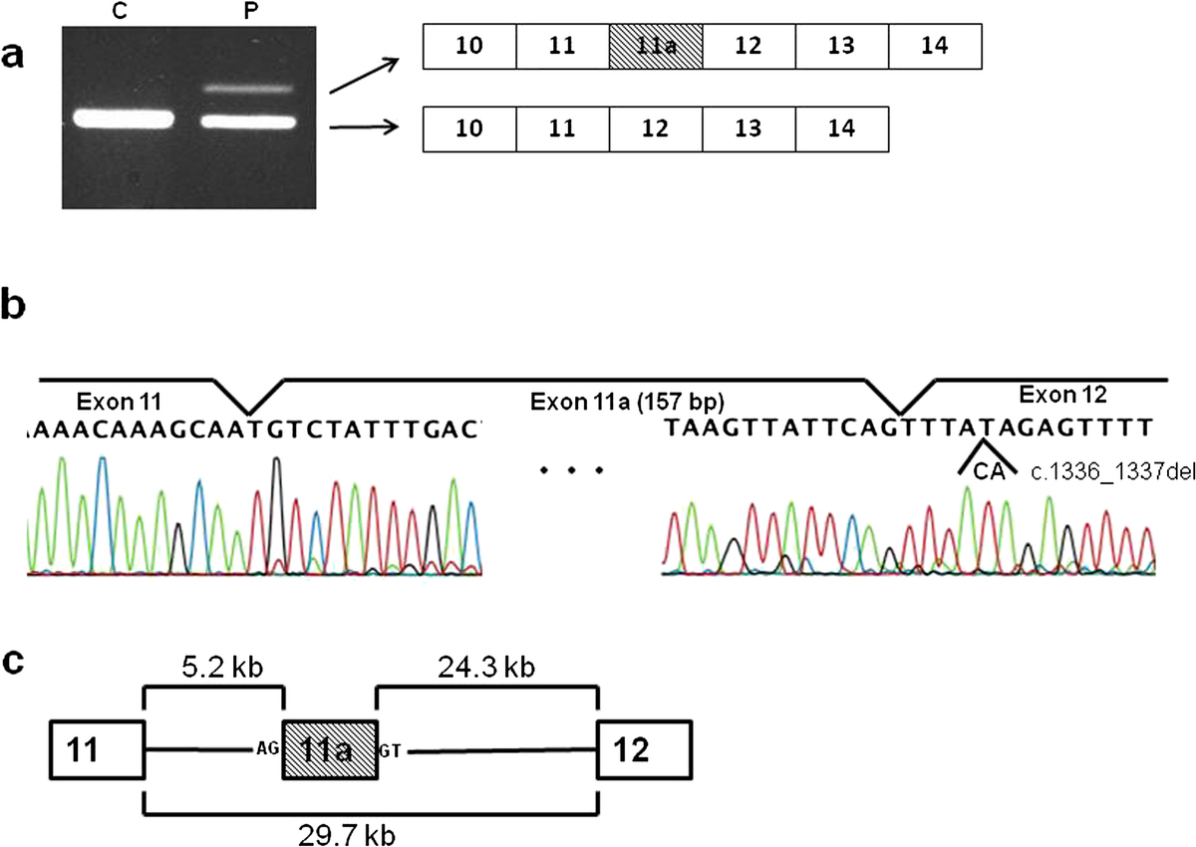
**Cloning of cryptic exon 11a**. a. RT-nested PCR products. A fragment spanning exons 10 to 14 was amplified by RT-nested PCR. Two amplified products were obtained from peripheral lymphocytes of a DMD patient (P) but not a control (C). A schematic representation of the exon structure of the amplified fragments is shown on the right. b. Sequences at the exon junctions. Subcloning and sequencing of the amplified products revealed that the larger product contained a 157-bp insertion (exon 11a) between exons 11 and 12. c. Schematic description of the location of exon 11a. The 5' and 3' ends of exon 11a (hatched box) are 5.2 kb downstream of exon 11, and 24.3 kb upstream of exon 12, respectively. Both the AG and GT splicing consensus dinucleotides are present adjacent to exon 11a.

We evaluated the decision tree by analyzing a novel cryptic exon 11a that was identified in this study. Exon 11a was found inserted into *dystrophin *mRNA in one DMD case who had a two-nucleotide (CA) deletion at the 5th and 6th nucleotides of exon 12 (c.1336_1337del). RT-nested PCR amplification of a fragment spanning exons 10 to 14 from this individual revealed two products: one corresponding to the normal size and the other larger than expected (Figure 2). Sequencing of the two products revealed that the normal-size band had the predicted exon content with the two-nucleotide deletion, and the larger product contained a 157-bp unknown insertion between exons 11 and 12. The inserted 157-bp sequence was identical to part of the 29.7-kb-long intron 11. The corresponding intronic sequence was present 5244 nt downstream of the 3' end of exon 11 and 24,278 nt upstream of exon 12 (Figure 2c). The inserted sequence maintained the AG and GT dinucleotide consensus sequences for splicing acceptor and donor sites at either end. Although the sequences flanking the inserted sequence were examined in this individual, no nucleotide change was found. The inserted sequence was named exon 11a.

**Figure 3:**
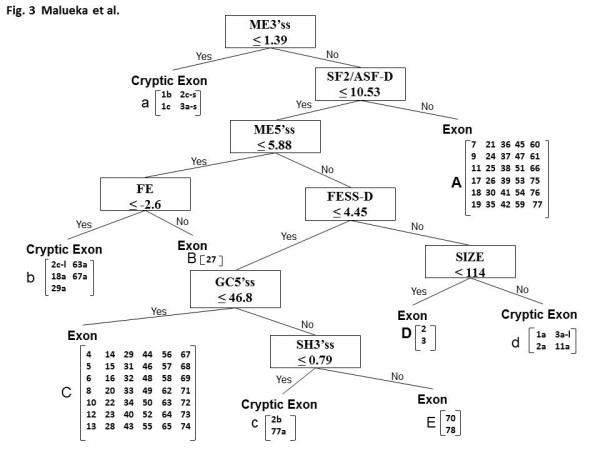
**Final decision tree to classify 77 authentic and 15 cryptic *dystrophin *exons**. The structure of the tree is as described for Figure 1. The features tested in this tree include seven indexes used in the preliminary tree (Figure 1) and one additional index, exon size (SIZE). This tree classified the authentic *dystrophin *exons into five groups (groups A to E), containing 30, 1, 42, 2, and 2 exons, respectively. The cryptic exons were classified into four groups (groups a to d).

When we tested exon 11a on our decision tree, it was classified not as a cryptic exon but as a real exon. Therefore, we decided that this tree was not suitable to classify exon 11a. We reconstructed the decision tree, including exon 11a as an additional cryptic exon using the same 26 indexes that were used to construct the first decision tree. The final tree, which had eight nodes, was able to separate all 15 cryptic exons from all 77 authentic exons with 100% accuracy (Figure 3). ME3'ss was the first splitting variable, with a cut-off point of 1.39. At this node, four cryptic exons were classified into one group (group a; Figure 3). SF2/ASF-D was the splitting variable at the second node, with a cut-off point of 10.53. A group of 32 exons (group A) was categorized on its "no" branch. Group A, therefore, was characterized by a high-density of ESEs recognized by SF2/ASF. At the third node, ME5'ss with a cut-off point of 5.58 was used to divide the data into two subnodes. On the "yes" branch, FE with a cut-off point of -2.6 split the group: one exon group consisting of five cryptic exons (group b) and one exon group containing only exon 27 (group B). On the other, "no" branch, FESS-D with a cut-off point of 4.45 divided the group into two subnodes. On the "yes" branch, GC5'ss with a cut-off point of 46.8 was used at the fifth node. One big exon group (group C), comprising 42 exons, formed the "yes" branch. On the other, "no" branch, SH3'ss with a cut-off point of 0.79 was used at the sixth node, generating a cryptic exon group (group c) and a group containing two authentic exons (exons 70 and 78; group E). On the "no" branch at the fourth node, SIZE with a cut-off point of 144 was next used, categorizing the final data into one exon group consisting of exons 2 and 3 (group D) and one group containing four cryptic exons (group d).

We evaluated our re-constructed tree using nine disease-causing exons that have been identified in the *dystrophin *mRNAs of dystrophinopathy patients. These pathological exons are created by the effect of deep-intron single nucleotide mutations. These mutations confer the characteristics of an authentic exon on a portion of intronic sequence, and thus the intron segment becomes recognized as an exon during the splicing process. Therefore, the decision tree should categorize these pathological exons as authentic exons. Remarkably, all nine pathological exons were correctly categorized into one of the four exon groups: four in group A, three in group C, and one in each of groups D and E. Therefore, we consider our decision tree a suitable classifier of pathological *dystrophin *exons.

Seven indexes were used in common by both the preliminary and re-constructed trees. However, their values in each tree were different, except for one (≤ 1.39 ME3'ss score at the first node). When we compared the two trees, we found that three exons (2, 70, and 78) and two exons (40 and 56) were re-categorized into small groups (groups E and D) and the largest group (group C), respectively. This indicated slight changes in the categorizing pathways between the two trees. The cryptic exons were ultimately categorized into four groups, and exon 11a belonged to group d, which comprises exons 1a, 2a, and 3a-l. It was interesting that group D was categorized by a SIZE score of ≤ 114 (Figure 3). This indicates that a small exon can be real if the splicing silencer density is high.

Among the 26 indexes used as candidates for the decision markers, only eight were used in the tree. Furthermore, five of these eight were those determining the strength of splice sites (Table 1). This indicates that recognition of the splicing sites is very important for proper splicing. Group E (exons 70 and 78) was categorized through six nodes; the final one discriminating it from group c (cryptic exons 2b and 77a). For this categorization, the strength of the 3'ss was tested twice using different indexes (ME3'ss and SH3'ss). This indicates the importance of the 3'ss strength relative to the 5'ss strength in *dystrophin *splicing. Indexes of ESE density (SF2/ASF-D) and ESS density (FESS-D) were also used in the tree. This indicates that both the ESE and ESS densities are important in the splicing process.

**Table 1:** Summary of categorization of exons by the decision tree

		Number of Exon	Indexes							
			
			ME3'ss	SF2/ASF-D	ME5'SS	FE	FESS-D	GC5'ss	SH3'ss	SIZE
**Group**	**A**	30	**H**	**H**	-	-	-	-	-	-
	
	**B**	1	**H**	**L**	**L**	**H**	-	-	-	-
	
	**C**	42	**H**	**L**	**H**	-	**L**	**L**	-	-
	
	**D**	2	**H**	**L**	**H**	-	**H**	-	-	**L**
	
	**E**	2	**H**	**L**	**H**	-	**L**	**H**	**H**	-

Results of categorization of 77 *dystrophin *exons are tabulated. Indexes determining the strength of splice sites are marked bold. H and L represent high and low at each node, respectively. Bars indicate not applicable.

Next, we used the tree to characterize exons that are subjected to specific splicing patterns. First, we marked exons known to be alternatively spliced (data not shown). However, they were found in all five groups. This indicates that no particular *cis*-elements predispose to alternative splicing. Second, we examined exons that were prone to splicing errors caused by intra-exon mutation. We have previously reported nonsense mutation-induced exon skipping in seven exons (exons 14, 15, 17, 27, 41, 42, and 70) [16]. Among these, only four (exons 17, 27, 41, and 42) cause disruption of an ESE. Remarkably, exons 17, 41, and 42 were categorized in group A, while all those that do not disrupt an ESE were categorized in other groups. This indicates that, for exons in group A, nonsense mutation-induced exon skipping is caused by disruption of an ESE, while for exons in other groups, nonsense mutation-induced exon skipping is caused by other factors. Third, we examined exons that splice to tissue-specific exon 1 (exons 2, 30, 45, 56, and 63). We did not identify any particular categorization characteristics for these exons.

## Discussion

The *dystrophin *gene produces many mRNAs because it is so complex: it contains 79 exons, huge introns, eight tissue-specific promoters, 15 cryptic exons, and generates many alternatively spliced products. It has been reported that there is a gradient in exon and intron definition at the level of pre-mRNA splicing [17]. For example, efficient use of intrinsically weak cryptic splice sites in exons is facilitated by a higher-than-average density of ESSs and a high density of SF2/ASF ESE motifs [17]. This suggests that each exon has a specific splicing regulatory mechanism in which particular splicing factors could compensate for the lack of other splicing factors. It is important to understand more about the splicing regulatory mechanisms for each exon of the *dystrophin *gene because treatment with exon skipping is a promising therapeutic approach.

Decision trees have been used in many kinds of classifications, such as for the management of Parkinson's disease, disease severity profiling, large-scale proteomic studies, and microarray data classification [14,18]. Here, we classified 77 authentic and 15 cryptic *dystrophin *exons into five groups based on their splicing regulatory factor characteristics. In this study, we needed to modify our preliminary decision tree (Figure 1) based on data from the novel exon 11a (Figure 2). This suggested a possibility that the tree needs further modification when another cryptic exon is identified in the future construction. However, we believe any modifications, if necessary, will be minimal as exon 11a was additionally analyzed (Figure 3). Moreover, our trees were constructed using indexes obtained only from *cis*-elements; further modification incorporating information from *trans*-elements may further improve the reliability of the tree.

We used our decision tree to determine the parameters that are most useful for the discrimination of authentic and cryptic *dystrophin *exons. We separated the two categories completely using eight variables: (1) strength of the 3'ss measured by maximum entropy score; (2) density of ESEs particularly SF2/ASF; (3) strength of the 5'ss measured by maximum entropy score; (4) free energy in U1-snRNP binding to the 5'ss; (5) density of ESSs as identified by FESS-D; (6) GC content at the 5'ss; (7) exon size; and (8) strength of the 3'ss as measured by the Shapiro score. In other words, the strength of the 3'ss consensus sequence as indicated by maximum entropy score is the most critical, as all authentic *dystrophin *exons must have a maximum entropy score of more than 1.39 at the 3'ss. The next most important deciding factor is ESE density, particularly for SF2/ASF; almost half (30) of *dystrophin *exons can be classified based on this parameter and 3'ss strength only. These findings are in line with those of a previous report, which showed that exons that are skipped because of splice site mutations have a weaker 3'ss and a lower-than-average density of ESEs [17].

Our exon categorization suggested that there are at least five different patterns of splicing regulatory mechanisms for *dystrophin *pre-mRNAs (Table 1). At the extreme, one group contained only one exon (exon 27; group B), implying that this exon has a unique splicing regulatory mechanism. In fact, alteration of exon 27 splicing has been reported for two nonsense mutations, resulting in a mild phenotype [19]. Exon 27 may be particularly vulnerable to splicing alterations caused by intra-exonic mutations.

On the other hand, two groups (A and C) contained 72 out of 77 exons, suggesting that most *dystrophin *exons are under similar splicing control. Group A, consisting of 32 exons, was characterized by a high density of ESEs, particularly SF2/ASF (more than 10.53) (Figure 3) (Table 1), indicating that ESE density plays a critical role in the splicing of these exons. Disruption of ESEs for exons in this group is more likely to cause exon skipping compared with disruption of ESEs in the other exon groups. Nonsense mutations within exons 17, 41, and 42, all belonging to group A, have been shown to induce exon skipping [16]. Accordingly, the nonsense mutations in these three exons disrupt an ESE sequence and thus cause exon skipping, while nonsense mutations that induce skipping of exons in other groups do not disrupt an ESE [16]. However, not all ESE-disrupting nonsense mutations identified in these exons induce exon skipping [16]. It may be necessary to consider *trans*-elements to explain these differences, along with the strength of splicing factor binding to the ESEs and their positions in the pre-mRNA secondary structure.

Intronic pseudo-exons that look like perfect exons, maintaining splicing consensus sequences, are now under intensive investigation [10,20-22]. There is evidence that inclusion of many of these sequences in mRNAs is actively inhibited because of the presence of intrinsic defects, the presence of silencer elements, or the formation of an inhibitory RNA secondary structure [23]. We categorized the cryptic exons into four groups (Figure 3), indicating a heterogeneous contribution of splicing factors required for their activation.

Group C contained 42 exons and was separated at the fifth node by a score of less than 46.8% GC content at the 5'ss. More than half of the *dystrophin *exons belonged to this group and are therefore presumed to be subject to similar splicing regulation. From the decision tree we can see that this group is characterized also by an ESS density ≤ 4.45 (FESS-D), indicating that splicing silencer signals play a critical role in the splicing of these exons. Indeed, we showed previously that nonsense mutation-induced exon skipping of exon 31 was caused by the creation of a splicing silencer-binding site for hnRNP A1 [13]. It has been reported that negative elements play important roles in distinguishing real splicing signals from the vast number of false-positive splicing signals [9].

Because the *dystrophin *gene is so complex, it has the potential to produce many alternatively spliced transcripts that are translated into protein variants [24]. However, studies on alternative splicing are limited [6-8]. In one study, Sironi et al. identified 16 alternative transcripts and examined splicing regulatory factors such as the 3' and 5' consensus values and exonic splicing enhancer scoring matrices; however, no reasonable explanation of the alternative splicing was identified [7]. This is consistent with our findings that the cryptic exons fell into five different groups. It is possible that alternative splicing does not rely completely on specific sequence elements and is regulated by *trans*-acting factors.

Interest in the splicing regulation of *dystrophin *pre-mRNA was first aroused when exon skipping caused by a small intra-exonic deletion was identified in a DMD patient [25]. Subsequently it was revealed that the deleted region was an ESE sequence within exon 19 [1] and that AOs against this ESE successfully induced the skipping of exon 19 [26]. This has led to the establishment of exon-skipping therapy [27]. In this study, we demonstrated that exon 19 was categorized into group A, characterized by a high ESE density; hence it is reasonable that AOs against the exon 19 ESE would induce exon skipping efficiently. Currently exon skipping is recognized as the most promising way to express dystrophin in DMD. The main targets for exon skipping therapy are exons 44, 45, 51, and 53 [28] and AOs against exon 51 are now in phase II or III clinical trials [2,3]. Because exons 45, 51, and 53 belong to group A, we would expect AOs against the ESEs in these exons to work well.

## Conclusions

The decision tree categorized the 77 authentic *dystrophin *exons into five groups. Our classification may help to establish the strategy for exon skipping therapy for DMD.

## Methods

### Indexes of splicing regulatory factors

The sequences of 14 known cryptic exons, and nine disease-causing mutations were obtained from our previous report [10] and the literature [29,30]. Twenty six indexes of splicing regulatory factors of each exon were obtained as described below (Table 2).

**Table 2:** Indexes of splicing regulatory factors

No	Features	Symbol	Reference
1	5' splice site strength (Shapiro score)	SH5'ss	[31]

2	3' splice site strength (Shapiro score)	SH3'ss	[31]

3	5' spice site strength (maximum entropy)	ME5'ss	[32]

4	3' spice site strength (maximum entropy)	ME3'ss	[32]

5	5' splice site strength (information content/Ri)	Ri5'ss	[33]

6	3' splice site strength (information content/Ri)	Ri3'ss	[33]

7	U1 SnRNA binding free energy	FE	[35]

8	ESE density (RESCUE ESE/RESE)	RESE-D	[36]

9	ESE density (PESE)	PESE-D	[38]

10	ESS density (FAS-ESS/FESS)	FESS-D	[37]

11	ESS density (PESS)	PESS-D	[38]

12	SF2/ASF number	SF2/ASF-N	[39]

13	SF2/ASF (IgM/BRCA1) number	SF2/ASF (IgM, BRCA1)-N	[39]

14	SRp40 number	SRp40-N	[39]

15	SC35 number	SC35-N	[39]

16	SRp55 number	SRp55-N	[39]

17	SF2/ASF score density	SF2/ASF-D	[39]

18	SF2/ASF (IgM/BRCA1) score density	SF2/ASF (IgM-BRCA1)-D	[39]

19	SC35 score density	SC35-D	[39]

20	SRp40 score density	SRp40-D	[39]

21	SRp55 score density	SRp55-D	[39]

22	5' splice site pre-mRNA secondary structure free energy	RSS5'ss	[40]

23	3' splice site pre-mRNA secondary structure free energy	RSS3'ss	[40]

24	5' splice site GC content	GC5'ss	[40]

25	3' splice site GC content	GC3'ss	[40]

26	The number of nucleotides in exon	SIZE	

#### Splice site strength

Splice site strength was determined in three ways: Shapiro's splicing probability matrix scores at the 5'ss and the 3'ss were calculated using published formulae [17,31] (SH5'ss and SH3'ss, respectively). Maximum entropy scores at the 5'ss and the 3'ss were obtained using online tools available at http://genes.mit.edu/burgelab/maxent/Xmaxentscan_scoreseq.html (ME5'ss and ME3'ss, respectively) [32]. Information content values at the 5'ss and the 3'ss were obtained using the Delila server at https://splice.uwo.ca/ (Ri5'ss and Ri3'ss, respectively) [33].

#### Free energy of U1 snRNA binding to the 5'ss

U1 snRNA binding to constitutive splice sites has lower free energy than that of its binding to alternatively spliced exon splice sites [34]. Free energy was analyzed using the SROOGLE server at http://sroogle.tau.ac.il/ (FE) [35].

#### Numbers and densities of ESEs and ESSs

The number of ESEs in each exon was calculated using the prediction algorithm at http://genes.mit.edu/burgelab/rescue-ese/ (RESE). The number of ESSs was calculated using two algorithms: at http://genes.mit.edu/fas-ess/ (FESS) and http://cubweb.biology.columbia.edu/pesx/ (PESS) [17,36-38]. To calculate the densities of ESSs/ESEs, the RESE, FESS, and PESS numbers were divided by the sequence length (in nucleotides) and this figure was multiplied by 100 [17] to give the RESE-D, FESS-D, and PESS-D scores.

The numbers of binding sites for the four SR proteins (SF2/ASF, SRp40, SC35, and SRp55) were obtained using ESEfinder (v. 3.0) available at http://rulai.cshl.edu/cgi-bin/tools/ESE3/esefinder.cgi?process=home (SF2/ASF-N, SRp40-N, SC35-N, and SRp55-N) [39]. The density of SR protein-binding sites (SF2/ASF-D, SC35-D, SRp40-D, SRp55-D) was obtained by dividing each number by its nucleotide length then multiplying it by 100 [17].

#### Minimum free energy value of pre-mRNA secondary structure and GC content around splice sites

Minimum free energy values of the pre-mRNA secondary structure at the 5'ss and 3'ss were obtained using the free energy minimization program RNAfold http://rna.tbi.univie.ac.at/cgi-bin/RNAfold.cgi using the 70 nucleotides both up- and down-stream of each splice site (RSS5'ss and RSS3'ss, respectively) [40,41].

GC content around splice sites has been reported to affect splicing [40]. Thus, the percentage of GC content for each 70 nucleotides both up- and down-stream of the 5'ss and the 3'ss was calculated (GC5'ss and GC3'ss, respectively) [40].

#### Exon size

The number of nucleotides in each exon was also taken into account (SIZE).

## Construction of decision trees

The C4.5 algorithm was used to construct decision trees. The C4.5 decision tree algorithm is an approach for pattern recognition and data mining [42,43] and was developed by Quinlan [44,45]. The algorithm uses information gain, which is an entropy-based criterion in information theory for decision tree construction. In this study, the conditions were as follows: (1) at least one object was to be contained in each branch, (2) the pruning confidence level was set to 100%, and (3) the iterative mode was used to avoid a local optimum.

We used 92 out of 94 data points (79 exons and 15 cryptic exons) for decision tree construction. We excluded two exons, exons 1 and 79, because they lacked some feature values.

### *Dystrophin *mRNA analysis

A 1-year-old Japanese boy (KUCG 952) without any family history of neuromuscular disorders was referred to Kobe University Hospital (Kobe, Japan) because of a high serum creatine kinase level (37,110 IU/l). DMD was tentatively diagnosed, and mutation in the *dystrophin *gene was analyzed after obtaining informed consent from his parents. *Dystrophin *mRNA expressed in his peripheral lymphocytes was analyzed as described previously [10,25]. A fragment spanning exons 10 to 14 was amplified by reverse transcription (RT)-nested PCR using two sets of primers. The outer primer set comprised a forward primer, 1A 5'-TTTTTATCGCTGCCTTGATATACA-3' and a reverse primer, 1B 5'-ACTCTGCAACACAGCTTCTGAG-3'; the inner primer set comprised a forward primer, 1E 5'-TTGCAAGCACAAGGAGAGATT-3' and a reverse primer, c14r 5'-ACGTTGCCATTTGAGAAGGAT-3'. The amplified fragments were resolved by agarose gel electrophoresis. Sequencing of the amplified products was performed by subcloning sequencing as described previously [46].

The mutation study was approved by our ethical committee and mutation analysis of the *dystrophin *gene was done after obtaining the informed consent from the parents of the patient.

## Abbreviations

DMD: Duchenne muscular dystrophy; AO: Antisense oligonucleotide; ESE: Exonic splicing enhancer; ESS: Exonic splicing silencer; ME3'ss: Maximum entropy of 3' splice site; SF2/ASF-D: SF2/ASF score density; ME5'SS: Maximum entropy of 5' splice site; FE: Free energy of U1 SnRNA binding; FESS-D: Fluorescence-activated screen-for exonic splicing silencers density; GC5'ss: GC content of 5' splice site; SH3'ss: Shapiro score of 3' splice site; SIZE: Size of exon.

## Competing interests

The authors declare that they have no competing interests.

## Authors' contributions

RGM carried out the molecular genetic studies, participated in the bioinformatics analysis and drafted the manuscript. YT carried out the bioinformatics analysis. MY, HA, TL and EKD participated in the clinical and genetic analysis. YT participated in the design of the study. MM conceived of the study, and participated in its design and coordination and helped to draft the manuscript. All authors read and approved the final manuscript.
